# Rethinking the neonatal transport ground ambulance

**DOI:** 10.1186/s13052-019-0686-y

**Published:** 2019-08-07

**Authors:** Carlo Bellini, Martina de Biasi, Maurizio Gente, Luca A. Ramenghi, Roberto Aufieri, Diego Minghetti, Silvia Pericu, Martina Cavalieri, Niccolò Casiddu, on behalf of the Neonatal Transport Study Group of the Italian Society of Neonatology (Società Italiana di Neonatologia, SIN)

**Affiliations:** 1 Neonatal Emergency Transport Service, Neonatal Intensive Care Unit, Department Mother & Child, IRCCS Gaslini Institute, Genoa, Italy; 2 Department of Architecture and Design (DAD), University of Genoa, Genoa, Italy; 3 Neonatal Emergency Transport Service, Maternal Infant Department, Policlinico Umberto I, Sapienza University of Rome, Rome, Italy; 4 Division of Neonatology and Neonatal Intensive Care, ASL Roma 2 - Ospedale Sant’Eugenio, Rome, Italy

**Keywords:** Neonatal transport, Ground ambulance, EU rules

## Abstract

**Objectives:**

This article describes the detailed project aimed to realize a dedicated ground ambulance for neonatal emergency transport service (NETS). To date, the European Community rules specify requirements for the design, testing, performance, and equipping of road ambulance used for transport and care of adult injured or ill patients, completely ignoring neonatal transport.

**Methods:**

The project consisted of electric and gas supply planning, interior design taking into account ergonomic and occupant protection principles, both during travel and during medical care performances.

**Results:**

A detailed project is presented. Main differences between European Type C ambulance and the new proposed Type D neonatal ground ambulance are the presence on board of air compressed cylinder, iNO cylinders and delivery system, phototheraphy, shock adsorbing stretcher support, cooling device, patient’s placenta (refrigeration box), and transcutaneous gas analyzer.

**Conclusion:**

The European Community rules specify requirements for road ambulance used for transport and care of adult injured or ill patients, completely ignoring neonatal transport.

This study describes the detailed project aimed to realize a dedicated ground ambulance for neonatal emergency transport service. This study demonstrated that it is not possible simply to adapt the currently dedicated ambulance for mobile intensive care and resuscitation services (actual type C European Community) in a modern dedicated NETS ambulance; it is of paramount importance suggesting to European Community to introduce a further ambulance type, to be identified type D, strictly reserved to neonatal transport activities.

## Introduction

The document EN 1789:2007 + A2:2014 ([1], see cross references inside) is issued by the European Union (EU) and specifies requirements for the design, testing, performance, and equipping of road ambulance used for transport and care of injured or ill patients, including requirements for the patient’s compartment. The described standards is applicable to road ambulances transporting at least one patient on a single stretcher and it is periodically revised, the most recent version being KKK-1822-F. What is now available in Europe is that the vehicle classification is standardized while the crew and its abilities are not yet. European ground ambulance are classified as Types A1 and A2, then type B and C. In particular, Type C ambulance is used in emergency setting, providing mobile intensive care and resuscitation services. The list of needed equipments is really very long and is described in detail into the document. Although this recently proposed classification represents a significant improvement, nothing is specified with regard to neonatal transport dedicated ambulance, that, at moment, are still ignored by EU. The documents EN 13976–1 and EN 13976–2 [2, 3] describe in detail the rescue systems and transportation incubators, with regard to interface conditions and system requirements, respectively. Surprising, these two documents focused on items in order to improve fixation, interchangeability and interoperability of the transport incubator using different ambulances and aircrafts, but excluded suggestions on standards for stretchers, vehicles or medical devices.

The aim of this article is to suggest a project to realize a dedicated ground ambulance for the neonatal transport, which function must be strictly linked to the on-board presence of a neonatal transport incubator which is designed and manufactured for specific purposes; then, to modify the type C ambulance design and suggesting to introduce the actually lacking European Union type D ground ambulance, eventually to dedicate exclusively to neonatal transport services.

## Matherial and methods

The Article is derived from an Architecture Product Design Degree Thesis (M. De Biasi) entitled “Redesign of the Ambulance of the Neonatal Emergency Transport Service of the Gaslini Institute, Genoa”, presented in October 2018, University of Genoa, Italy. We planned to create a task force between product designers from the Department of Architecture and Design (DAD) of the University of Genoa, Italy, the Neonatal Emergency Transport Service (NETS), Gaslini Institute Children’s Hospital, Genoa, and the Neonatal Transport Study Group of the Italian Society of Neonatology, Italy. The choice of the vehicle was for a van type, conforming to the commonly used ambulance vehicle in Europe; we then select to base our project on the Fiat Ducato van, being the most used vehicle in Italy and one of the most used in Europe as ground ambulance. Our NETS actually uses a Fiat Ducato too, thus easing our project. We selected the version Ducato Van Glazed / Semi-glazed in order to have a large lateral glass surface available to improve the NETS team travel comfort. The project consisted of electric and gas supply planning, interior design taking into account ergonomic and occupant protection principles, both during travel and during medical care performances.

## Results

**Fig. 1:**
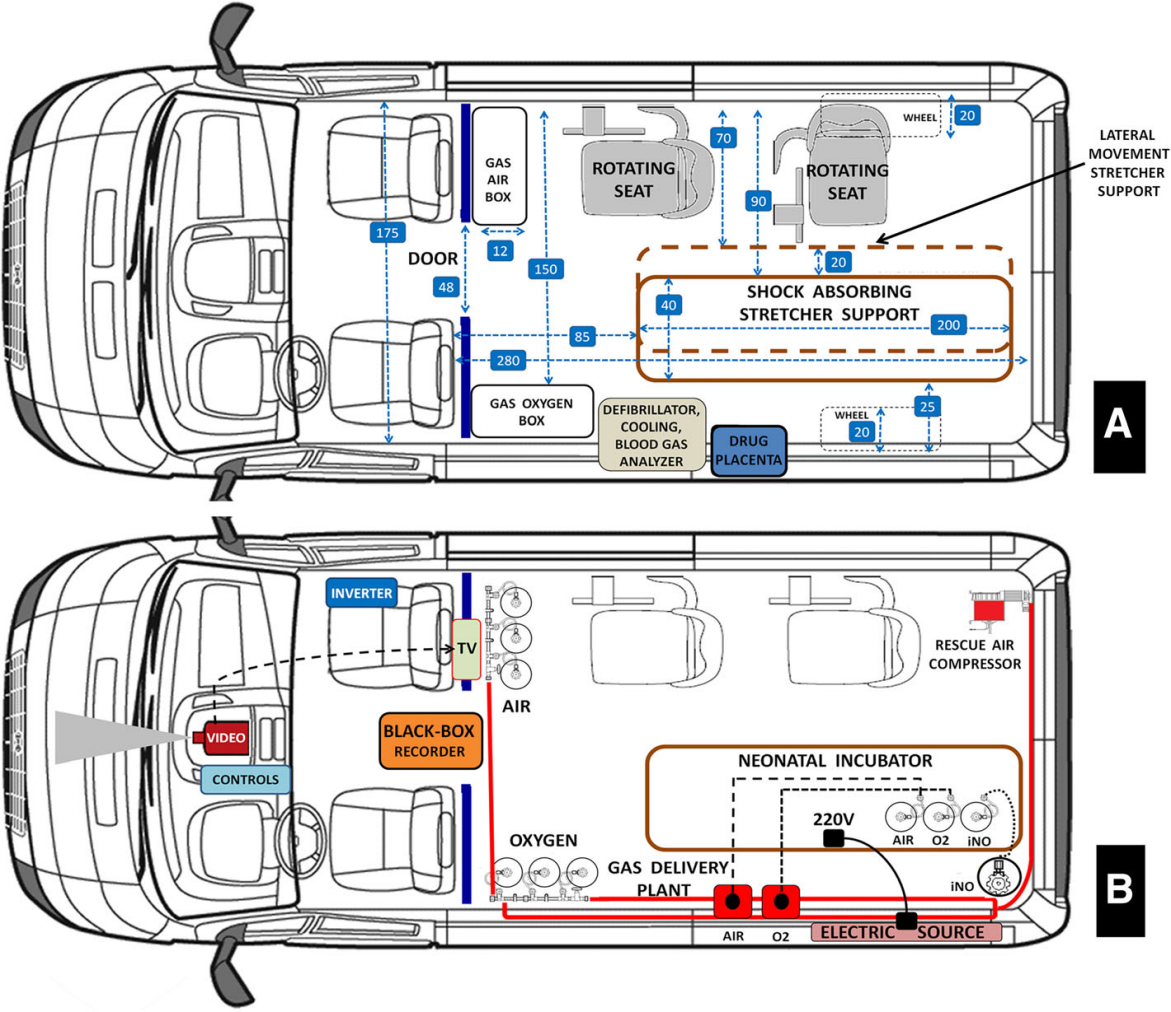
Aerial floor plan projection. Panel **a**, interior design. Panel **b**, electric and gas pipeline system. Panel **a**. The right side is fitted with two rotating seats for neonatal transport team members; seats can rotate 90° towards the left. In front of the seats there is the box containing three oxygen cylinders (10 l/200 bars each); a stretcher and incubator support is fixed to the ambulance floor along the longitudinal axis of the health compartment; this device is equipped with shock absorber featuring efficient hydropneumatic system to reduce the effects of vibrations and impact during long transfers or transport on rough terrain. The support can move laterally to the right by 20 cm, allowing to place it near the rotating seats. The left ambulance wall unit is composed by the boxes containing three air compressed cylinders (10 l/200 bars each), then the neonatal defibrillator, the device for active controlled patient cooling, the blood gas analyzer, and finally the two separated refrigerated boxes to contain drugs and the transported patient’s placenta. Numbers into the square box indicate ambulance dimensions in centimeters. Red emergency transport bag is fastened to the ambulance floor, thus being immediately available (not shown in figure). Panel **b**. Medical gases (oxygen and medical air) are distributed throughout the ambulance via the pipeline distribution system to provide gases at the terminal units. Socket panel for medical gas is provided by valves and pressure gauges. A separated vacuum pipeline is available also. In the rear right part of the ambulance an air compressor is fitted out; this is a rescue device. An iNO cylinder is stored in the left rear part of the ambulance and a second iNO cylinder is transported by the incubator. A dedicated iNO pipeline works independently by oxygen and air pipelines. Above the cylinder box, in front of seats, there is a television reproducing the video signals from the video-camera positioned on the ambulance cockpit.

**Fig. 2:**
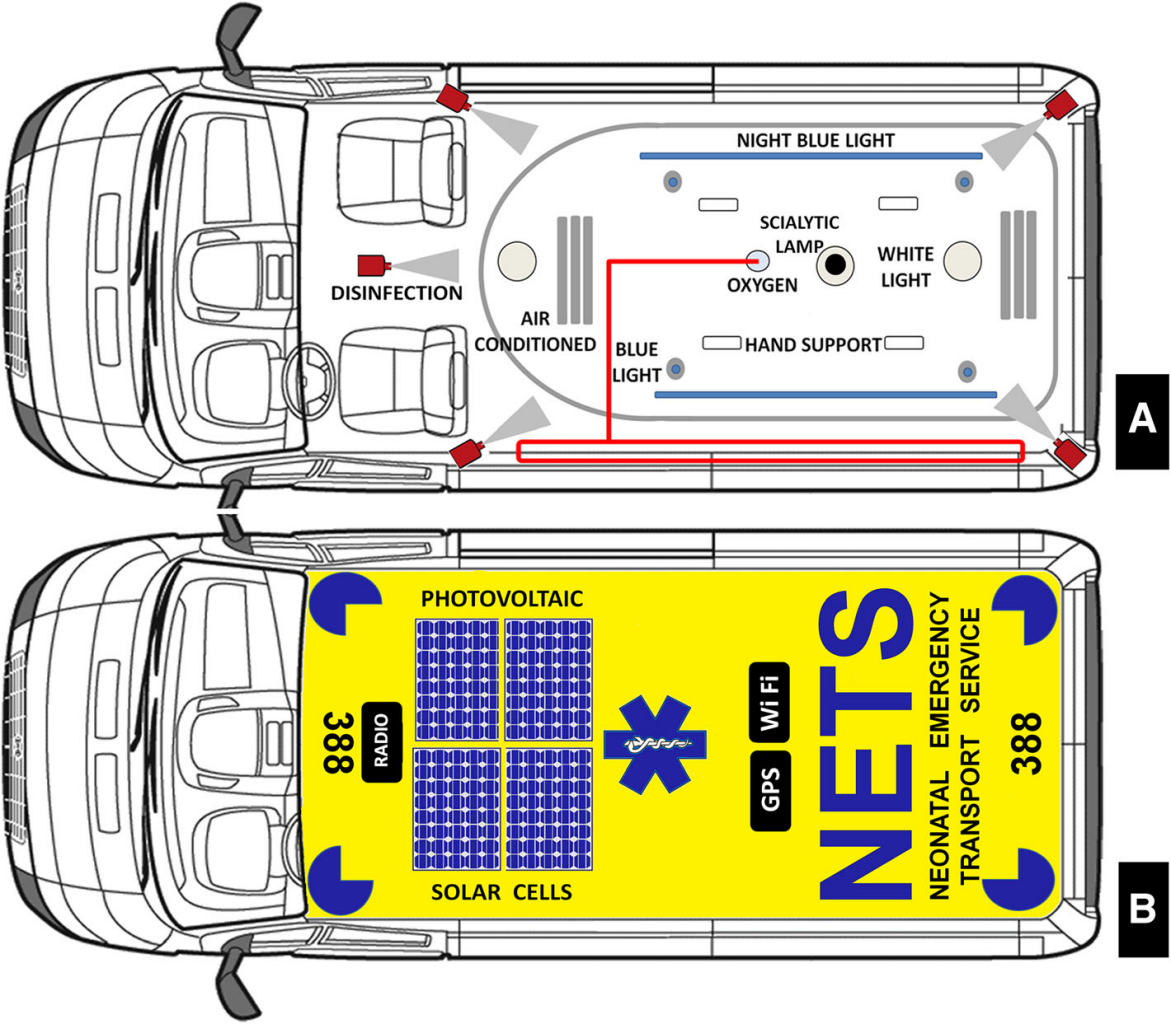
Ceiling and roof projection. Panel **a**, ceiling view. Panel **b**, roof section. Panel **a**. Hi-Lo led dome lights, night blue light, a scialytic lamp, an automatic disinfection system fitted with jets nebulizing nozzles are present. Both driver and health compartment are fitted with own heating and air-conditioning systems which can operated independently. Panel B. Two solar photovoltaic panels for rescue charging the vehicle batteries is activated by control units from the cockpit. Radio, GPS, and WiFi antenna and usual blue led flashing light and siren completed this section

**Fig. 3:**
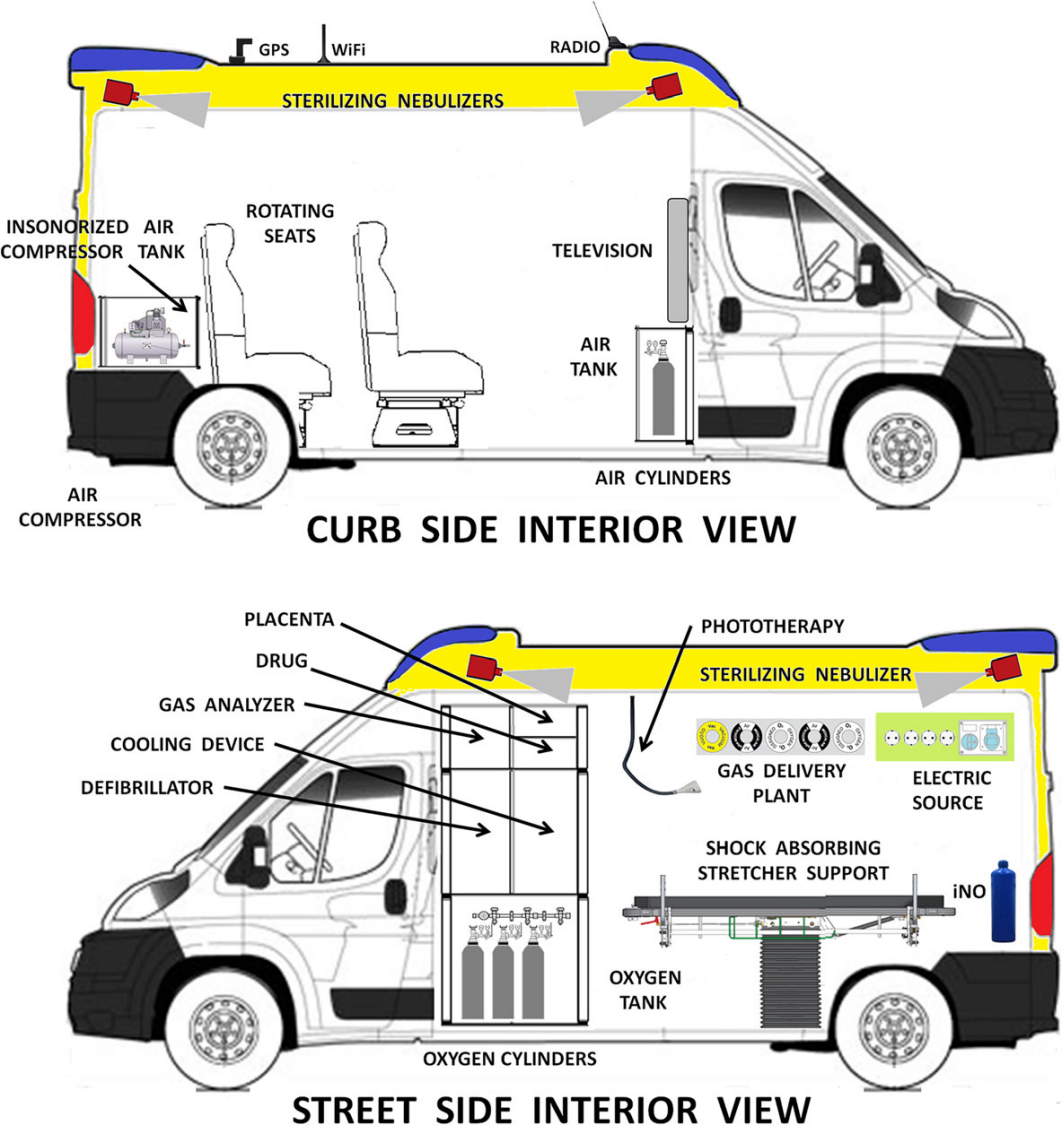
Curb and street lateral interior views. Phototherapy lamp is illustrated in this section

**Fig. 4:**
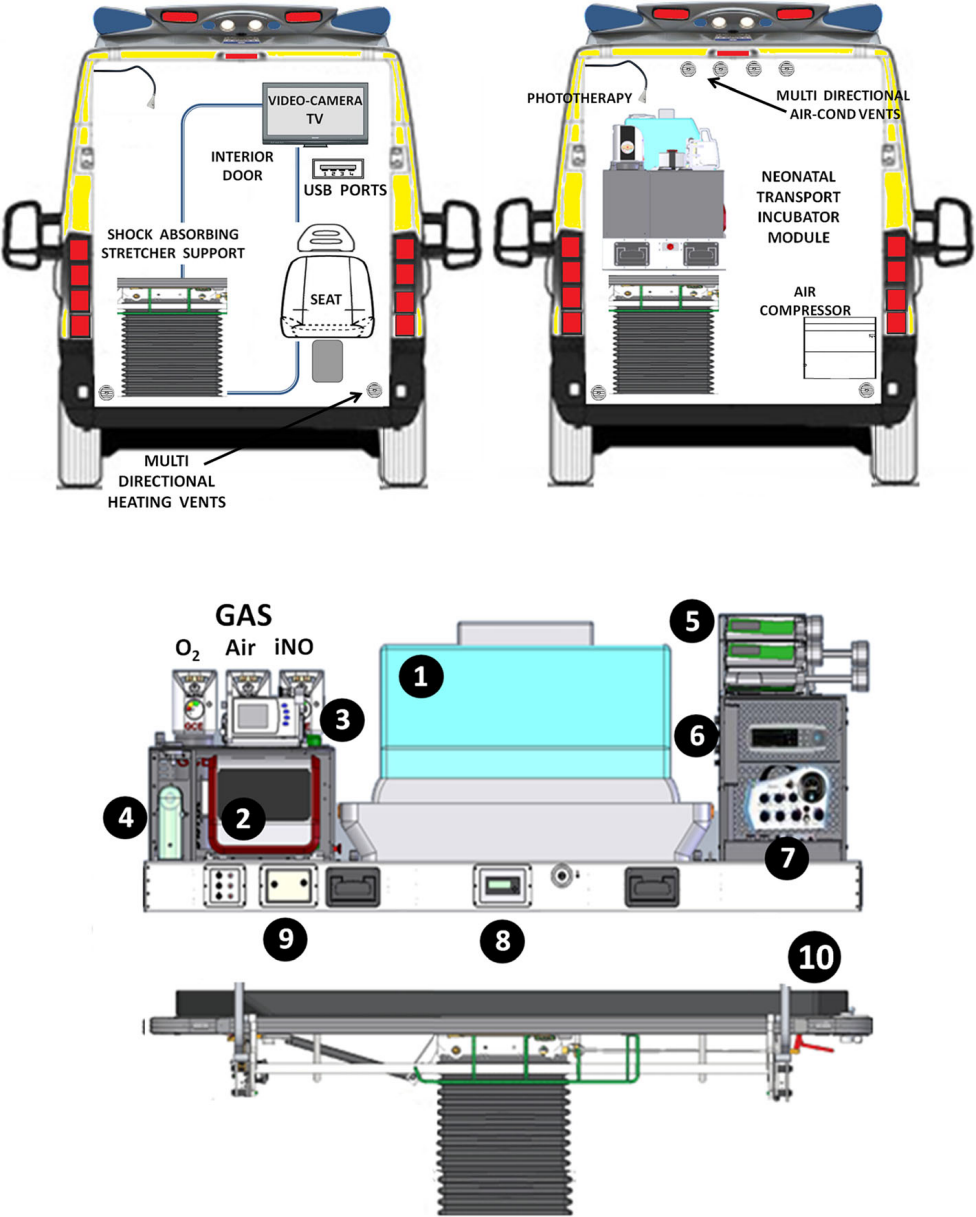
Forward interior view (above) and detailed neonatal transport incubator scheme (below). Right forward view shows the transport incubator mounted on the shock adsorbing stretcher support. The schematic design concerns to the neonatal incubator actually in use for our NETS. In detail: #1, incubator; #2 neonatal ventilator; #3 monitor; #4 suction device; #5 infusion pumps; #6 transcutaneous monitor; #7 s neonatal ventilator (twin transport) (8); #8 iNO monitor; # 9 transport heather humidification system; #10 shock adsorbing stretcher support. The neonatal transport module is provided of own gas cylinders

The results essentially coincide to the project description. Figures 1, 2, 3, 4 describe the ambulance project in a schematic form. Details of the project are reported in Figure legends. List of main differences between European Type C ambulance and previously unreported Type D ground ambulance are shown in Table 1

**Table 1:** Differences between Type C and D Ambulance

List of main differences between Type C and Type D ground ambulance		
Equipment	EU EN 1789 Type C ground ambulance	Our suggested Type D neonatal transport ground ambulance
Mechanical ventilator	Adult use only	Neonatal use only
Oxygen cylinders	Present	Present
Air compressed cylinders	Not planned	Needed
iNO cylinders and delivery system	Not planned	Needed
Phototheraphy	Not planned	Needed
Shock adsorbing stretcher support	Not planned	Needed
Second mechanical ventilator (twin newborn transport)	Not planned	Needed (mounted on transport module)
Defibrillator	Adult use only	Neonatal use only
Cooling device	Not planned	Needed
Patient’s placenta (refrigeration box)	Not planned	Needed
Transcutaneous gas analyzer	Not planned	Needed (mounted on transport module)

In general, we decided to avoid the installation of cabinets, usually assigned to contain various accessories for adult use, such as, for example, immobilizer and seat belts, vacuum splints, emergency seats, cervical collars, vacuum mattress, or other devices that are totally worthless during neonatal transport, as well as, cabinets possibly earmarked to contain essential features of equipment used in neonatal transport, such as glove and catheter box, intubation set material, or central catheters, that are usually stored into the transport bag, thus being immediately available and easily transportable.

## Discussion

Neonatal patients are routinely transported to tertiary care facilities, not infrequently over long distances; the quality of neonatal emergency transport systems (NETSs) evolved and improved during the last years worldwide, making actual NETSs like on-wheel moving NICU [3–6].

Since the beginning of NETS activities, it was clear that highly specialized personnel and appropriate equipment were essential. The limited market for transport equipment has resulted in homemade, customized and nonstandardized systems, eventually leading to equipment failure [5, 6]. Despite the enormous progress in planning ambulance for intensive care, and despite the reported usefulness to have available dedicated ambulance to neonatal transport service, to date specific rules describing the ideal ambulance for neonatal transport are still lacking [7–9]. Planning our project, we decided to avoid the installation of cabinets, usually assigned to contain various accessories for adult use, such as, for example, immobilizer and seat belts, vacuum splints, emergency seats, cervical collars, vacuum mattress, or other devices that are totally worthless during neonatal transport, as well as, cabinets possibly earmarked to contain essential features of equipment used in neonatal transport, such as glove and catheter box, intubation set material, or central catheters, that are usually stored into the transport bag, thus being immediately available and easily transportable. This choice allowed us to design a health compartment with a highly reduced number of kinematic mechanisms, protrusions or sharp corners, thus improving passive safety and increasing the usable space. The health compartment is equipped with waterproof floor with a perimeter edge of 50 mm, easily washable with water jet. In our project we do not consider mechanical characteristics of the vehicle; we underline that adjustable suspension to provide and assure comfortable ride is a fundamental tool, as well as, all safety equipments usually equipping modern vehicles. We believe that a strong point of our study is that we did not simply modify the EU type C ambulance for using it in neonatal transport activities, but, indeed, we designed the completely new EU type D ambulance previously not reported by EU documents. Adding to the EU type C ambulance equipment list the pipeline for nitric oxide use, the active controlled cooling device, the phototherapy lamp, the shock adsorbing stretcher support, and, obviously, a fully equipped neonatal transport module, definitively characterized the project (Table). The presence on board of a shock absorbing stretcher support, associated with an air-foam mattress and gel pillow, is very important for improving the safety and comfort of the transported patient reducing the effects of vibrations and impact during long transfers or transport on rough terrain [10]. Vans suitable to be arranged for ambulance use actually present on the market have all similar interior dimension that are approx one/third of the recommended standard of 25 square meters for NICU, thus making very important an accurate use of the available space. The possibility to move the stretcher support laterally to the right up to 20 cm and the presence of rotating seats allow the NETS team to work snugly and secure. Even though the longitudinal position of the stretcher support, our project established that the personnel can be seated facing the direction of travel, thus reducing motion sickness. Rotating seats and the 20 cm lateral movement of the incubator when fixed to the stretcher support allow one team member to sit at the head of the transported newborn to provide constant clinical monitoring and management of the airway while the second member can have continuous viewing of the monitor and easy access to additional equipment. A parent may be accommodate in the driver compartment, properly secured. The front video-camera can record the entire trip allowing NETS Direction to have available all the needed documentation to be used in case of accident possibly resulting in litigations, monitoring driver performance, and, at the same time, to provide to the NETS team in the back partially blinded health compartment to have on dedicated television a continuous view of the road and traffic, making personnel ready to take on drive emergency. We wish for equipping all neonatal transport ambulances with a dedicated black-box trip recorder to improve the safety level. The sterilizing nebulizer system is important to obtain a clear ambulance in short time. Although these plants are not exclusively for neonatal transport ambulance, the larger space obtained inside the health compartment removing useless devices and cabinets makes easily their assembly. The Resin Transfer Molding (RTM) allowed us to design a health compartment with a highly reduced number of kinematic mechanisms, protrusions or sharp corners, thus improving passive safety and increasing the usable space. Finally, the only cabinets we established to include are for, as reported above, neonatal defibrillator, cooling device, gas analyzer, and a refrigerator for drugs and, in a separated box, for another specific need of neonatal transport, i.e., the transport of the patient’s placenta. Radio, GPS, and WiFi antenna allow communications whether transporting, tracking patient’s vital sign, and secure transmission of photographs, medical images and electrocardiograms, when needed.

## Conclusion

The actual EU type C ambulance configuration is really far to the present proposed ambulance design to dedicate to neonatal transport; further, it is also clear that it is not effective simply to adapt the type C ambulance modifying it in a modern dedicate NETS ambulance, but, rather, that we need re-thinking and re-designing currently available ambulance model to eventually integrate the EU regulations adding the at moment absent type D ambulance, compliant to the present project, totally and exclusively dedicated to neonatal transport. Avoiding to fit an ambulance with superfluous devices for adult use, then adding specific equipment for neonatal transport use, is uselessly expansive; we are aware that it is not feasible to imagine the immediate replacement of the ambulances actually in use, but we hope that EU can accept our suggestions and that the future modern ambulances for NETS activities may be equipped taking into account the specific neonatal transport requirements. Further, we believe that this type D ambulance is cheaper than type C, or, at least, not more expansive due to the fact that many worthless devices for adult use only are excluded.

## Data Availability

Gaslini Institute, study office Dr. C. Bellini.
